# Measuring the improvement in health-related quality of life using King’s health questionnaire in non-obese and obese patients with lower urinary tract symptoms after alpha-adrenergic medication: a preliminary study

**DOI:** 10.1186/1471-2490-14-60

**Published:** 2014-08-06

**Authors:** Jae Heon Kim, Hoon Choi, Hwa Yeon Sun, Seung Whan Doo, Jong Hyun Yoon, Won Jae Yang, Byung Wook Yoo, Joyce Mary Kim, Soon-Sun Kwon, Eun Seop Song, Hong Jun Lee, Ik Sung Lim, Yun Seob Song

**Affiliations:** 1 Department of Urology, Soonchunhyang University College of Medicine, Seoul Hospital, 59, Daesagwan-ro, Yongsan-gu, Seoul 140-743, The Republic of Korea; 2 Department of Urology, Korea University College of Medicine, Ansan Hospital, Ansan, Korea; 3 Department of Family Medicine, Soonchunhyang University School of Medicine, Seoul, Korea; 4 International Clinic Center, Soonchunhyang University Hospital, Seoul, Korea; 5 Biomedical Research Institute, Seoul National University Bundang Hospital, Seongnam, South Korea; 6 Department of Obstetrics and Gynecology, Inha University School of Medicine, Incheon, Korea; 7 Medical Research Institute, Chung-Ang University College of Medicine, Seoul, Korea; 8 Department of Industrial Management and Engineering, Namseoul University College of Engineering, Cheonan, Korea

**Keywords:** Alpha-blocker, Prostatic hyperplasia, Body mass index, Waist circumference

## Abstract

**Background:**

The efficacy of medical treatment among obese men with lower urinary tract symptoms (LUTS) has been less clear, especially regarding the improvement of QoL. We aimed to investigate the difference in efficacy and consequent satisfaction of life quality after medical treatment of male LUTS according to obesity.

**Methods:**

An 8-week prospective study was performed for a total of 140 patients >50 years old with International Prostate Symptom Scores (IPSS) > 12 points and prostate volume > 20 mL. Obesity was determined by either body mass index (BMI) or waist circumference (WC). Patients were divided into 2 groups according to BMI or WC. Patients received tamsulosin at a dose of 0.4 mg daily for 8 weeks. The changes from baseline in the IPSS, maximal urinary flow rate (Qmax), post-void residual volume, questionnaire of quality of life (QoL), and King’s Health Questionnaire (KHQ) were analyzed.

**Results:**

Of the 150 enrolled patients, 96 completed the study. Seventy-five patients (78.1%) had BMI ≥ 23 kg/m^2^, and 24 (25.0%) had WC > 90 cm. Overall, the IPSS, IPSS QoL, and total KHQ showed significant improvement. Obese (BMI ≥ 23 kg/m^2^) and non-obese (BMI < 23 kg/m^2^) both showed improvement of the IPSS and IPSS QoL scores, but only the obese (BMI ≥ 23 kg/m^2^) group showed improvement of the total KHQ score (P < 0.001 vs. P = 0.55). Only the obese (WC > 90 cm) group showed improvement of the IPSS and total KHQ scores (P < 0.001).

**Conclusions:**

Our preliminary study showed the different efficacy of an alpha-blocker for improvement of LUTS and life quality according to obesity. Obese patients, defined by BMI or WC, showed the tendency toward a more favorable improvement of LUTS and life quality.

**Trial registration:**

Current Controlled Trials 2010–058. Registered 2 September 2010 in Soonchunhyang Univeristy Hospital

## Background

Benign prostatic hyperplasia (BPH)/lower urinary tract symptoms (LUTS) is a common disease entity in older men and has a negative impact on quality of life (QoL) [1-3]. Obesity is also a common condition in older males and is related to LUTS [1,3]. As in Western countries, Korean men have a high prevalence of symptomatic BPH as they age, with a prevalence of 10.6%–31% in men > 50 years old [2,4]. Several studies have reported a significant relationship between LUTS and obesity [3,5,6]. Considering the objective evidence of a negative effect of both LUTS [7-13] and obesity [14] on QoL, LUTS plus obesity may result in a greater deterioration of QoL.

Among the alpha blockers, tamsulosin is one of the most commonly recommended because of its efficacy, safety, and tolerability [15]. The King’s Health Questionnaire (KHQ) is a validated tool to measure the QoL in patients with LUTS [9]. Although the KHQ was developed originally for female urge incontinence, validity has been proven in studies that dealt with LUTS and health-related quality of life (HRQoL) in both sexes [9,16-19].

To date, efficacy of medical treatment among obese men with LUTS has been less clear, especially regarding the improvement of QoL within specific domains. Recently, Lee et al. [14] reported that alpha-blockers have a greater efficacy in improvement of LUTS when used in obese men. The main presumed hypothesis for this difference is that obese men have a greater level of sympathetic activity, which is suspected to be sensitive to alpha-blockers [20,21].

The main purpose of this preliminary study is to investigate the improvement of LUTS and QoL in different groups (obese and non-obese).

## Methods

### Study design

An eight-week prospective study was conducted between March 2010 and May 2011. The study was approved by the institutional review board of Soonchunhyang University Hospital.

At the initial visit, anthropometric parameters were evaluated, including weight, height, and waist circumference. Additional parameters measured included urinalysis, serum prostate-specific antigen [22], maximal urinary flow rate (Qmax), and post-void residual volume (PVR). Transrectal ultrasonography was also performed. At the initial and final visits, the International Prostate Symptom Score (IPSS) and KHQ were measured.

BMI was calculated as the body weight in kilograms divided by the square of the height in meters. After the first evaluation, eligible patients were treated with tamsulosin at a dose of 0.4 mg for 8 weeks. At each visit, adverse events were recorded.

Serum PSA tests were performed using the automated chemiluminescent microparticle immunoassay analyzer Architect i2000 (Abbott Diagnostic Laboratories, Abbott Park, IL, USA). The prostate was measured in three dimensions by transrectal ultrasonography using an 8.0-MHz rectal probe (GE Healthcare, LOGIQ P6-PRO, Little Chalfont, UK), and prostate volume (PV) was estimated using a modification of the prolate ellipsoid formula and recorded in cm^3^ (0.523 [length (cm) × width (cm) × height (cm)]).

Acquisition of questionnaire data was performed with face-to-face interviews conducted by one investigator with all study participants using a structured questionnaire. The severity of LUTS was measured using the IPSS, which is based on the American Urological Association [23] symptom index with one additional question on QoL, and is the most widely used objective assessment of LUTS.

The KHQ consists of 8 categories, including general health perceptions, impact on life, role limitations, physical/social limitation, personal relationships, emotions, sleep/energy, and incontinence severity measures. The KHQ has been validated in a Korean version [24].

### Subjects

A total of 150 patients who visited the Department of Urology at Soonchunhyang University and provided informed consent were included in this study. Inclusion criteria were male ≥ 40 years of age, no history of prior treatment of BPH/LUTS, total IPSS ≥ 8, prostate volume > 20 mL as determined by transrectal ultrasound, no relevant medical history, and underlying comorbidities. Exclusion criteria were history of neurogenic bladder dysfunction, history of prostate or bladder cancer, treatment history of acute or chronic urinary retention within the previous 3 months, treatment history of acute or chronic prostatitis within the previous 3 months, PSA levels > 0.10 ng/mL, and history of urinary tract infection or bladder stones.

All patients were divided into 2 groups, non-obese (BMI < 23 kg/m^**2**^) and obese (BMI ≥ 23 kg/m^2^), according to the Asia-Pacific obesity criteria [25]. For additional analysis, we also categorized patients into 2 groups based on waist circumference (WC), normal waist (≤90 cm) and central obesity (>90 cm).

### Power calculation

Sample size was not calculated because its being a preliminary study.

### Statistical analysis

The primary outcome measurement was the degree of change of KHQ in the two groups, and secondary outcome measurement was the degree of change of IPSS in the two groups. All data are presented as mean ± SD. Statistical analysis was performed using SPSS (version 21.0; Chicago, IL, USA). Changes from baseline in total IPSS, QoL scores, and KHQ scores were analyzed using Wilcoxon signed-rank test. A P value < 0.05 was considered significant.

## Results

**Figure 1:**
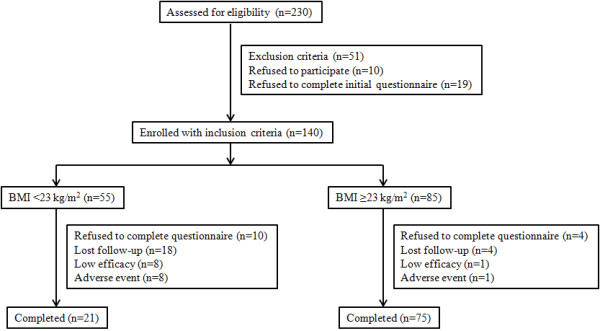
Flow chart of enrolled patients.

Of the 140 enrolled patients, 96 completed the study. Fifty four patients discontinued the trial. Of these, 22 were lost to follow-up, 14 refused to complete the final questionnaires, and 18 patients discontinued the medication because of low efficacy (9 patients) or adverse events (9 patients) (Figure 1).

The basic characteristics, including age, PSA, PV, Qmax, total IPSS, and KHQ questionnaire are described in Table 1. Between the obese group (BMI ≥ 23 kg/m^2^) and non-obese group (BMI < 23 kg/m^2^), no significant difference occurred among the variables. Between the central obesity group (WC > 90 cm) and non-central obesity group (WC ≤ 90 cm), no significant difference occurred among the variables except age.

**Table 1:** Basic characteristics of participants

	**BMI < 23**	**BMI ≥ 23**	**P value**	**WC ≤ 90**	**WC > 90**	**P value**
No	21	75		72	24	
Age	61.25±9.80	61.48±7.09	0.982	60.58±7.60	64.20±7.54	0.045
PSA	2.12±	2.04±	0.231	2.13±	1.98±	0.312
PV	31.12±10.14	34.23±8.71	0.042	32.31±9.81	33.23±10.21	0.218
Qmax	12.32±2.21	13.25±1.75	0.134	11.46±1.89	13.33±2.34	0.185
IPSS questionnaire						
Total IPSS	14.90±7.58	13.41±6.79	0.388	15.00±7.57	13.31±6.74	0.308
IPSS QoL	3.47±1.21	3.54±1.22	0.830	3.56±1.40	3.51±1.15	0.864
KHQ questionnaire						
General health	41.66±18.25	47.66±23.67	0.286	47.91±26.49	45.83±21.39	0.698
Impact on life	53.91±30.65	58.60±30.40	0.534	58.27±28.20	57.35±31.22	0.898
Role limitations	25.29±33.45	28.77±31.98	0.664	33.20±37.91	26.28±30.10	0.364
Physical limitation	29.24±31.45	27.44±30.08	0.811	33.20±34.26	26.05±28.79	0.318
Social limitation	22.19±31.81	16.71±25.48	0.411	18.03±24.83	17.87±27.59	0.980
Personal relationships	21.71±37.96	25.39±32.24	0.724	24.90±35.34	24.55±32.84	0.972
Emotions	25.90±31.06	27.52±25.83	0.808	31.91±29.15	25.59±26.12	0.321
Sleep/Energy	26.08±29.49	21.02±26.64	0.454	18.67±24.13	23.28±28.22	0.475
Incontinence	14.14±27.47	9.85±14.73	0.343	16.50±21.31	8.89±16.76	0.076
Total KHQ	251.88±223.71	254.88±171.03	0.947	274.31±198.90	247.53±178.02	0.537

After 8 weeks treatment with tamsulosin, overall improvement was seen in the total IPSS and IPSS QoL (P < 0.001) (Table 2). Total KHQ scores also improved after treatment (P < 0.001). Among the domains, general health, impact on life, role limitations, physical limitation, emotions, sleep/energy, and incontinence severity were improved or significantly improved. However, social limitation and personal relationships did not show improvement after treatment (Table 2).

**Table 2:** Differences in IPSS and KHQ before and after treatment

	**Mean difference**	**SD**	**95% CI**		**P value**
IPSS					
IPSS questionnaire	3.521	6.764	2.150	4.891	< 0.001
QoL	1.011	1.487	.701	1.321	< 0.001
KHQ					
General health	13.281	25.380	8.139	18.424	< 0.001
Impact on life	18.417	32.513	11.829	25.004	< 0.001
Role limitations	10.302	26.954	4.841	15.764	< 0.001
Physical limitation	9.490	26.417	4.137	14.842	0.001
Social limitation	3.323	24.418	-1.625	8.271	0.186
Personal relationships	5.283	24.118	-1.365	11.931	0.117
Emotions	9.656	19.902	5.624	13.689	< 0.001
Sleep/Energy	4.632	22.549	.038	9.225	0.048
Incontinence	3.010	14.141	.145	5.876	0.040
Total KHQ	76.167	144.346	46.920	105.414	< 0.001

In subgroup analysis, the non-obese group showed no improvement in total KHQ (P = 0.055) (Table 3). Among the KHQ domains, the obese group showed improvement only in general health (P = 0.025), impact on life (P = 0.020), and emotions (P = 0.047). The obese group showed improvement in total KHQ (P < 0.001) (Table 3). Among the KHQ domains, the obese group showed improvement in general health (P < 0.001), impact on life (P < 0.001), role limitations (P = 0.001), physical limitation (P < 0.001), and emotions (P < 0.001). The comparison of improvement of IPSS and KHQ revealed that obesity group showed significant greater improvement in total IPSS, physical limitation, and sleep/energy (Table 4).

**Table 3:** Differences in IPSS and KHQ before and after treatment according to BMI

	**BMI < 23**			**BMI ≥ 23**		
	**Mean difference**	**SD**	**P value**	**Mean difference**	**SD**	**P value**
IPSS questionnaire						
Total IPSS	5.571	7.928	.004	2.947	6.341	< 0.001
QoL	1.105	1.243	.001	.986	1.552	< 0.001
KHQ questionnaire						
General health	10.714	20.266	.025	14.000	26.712	< 0.001
Impact on life	20.619	37.377	.020	17.800	31.269	< 0.001
Role limitations	9.381	27.006	.127	10.560	27.116	0.001
Physical limitation	3.190	34.945	.680	11.253	23.483	< 0.001
Social limitation	8.429	27.770	.180	1.893	23.400	0.486
Personal relationships	5.556	37.250	.666	5.227	21.101	0.108
Emotions	10.571	22.892	.047	9.400	19.146	< 0.001
Sleep/Energy	7.048	25.488	.220	3.946	21.785	.124
Incontinence	6.524	23.159	.211	2.027	10.357	.094
Total KHQ	79.762	179.641	.055	75.160	134.246	< 0.001

**Table 4:** Differences in IPSS and KHQ before and after treatment according to waist circumference

	**WC < 90**			**WC ≥ 90**		
	**Mean difference**	**SD**	**P value**	**Mean difference**	**SD**	**P value**
IPSS questionnaire						
Total IPSS	2.667	7.191	.082	3.806	6.643	< 0.001
QoL	.957	1.637	.010	1.029	1.445	< 0.001
KHQ questionnaire						
General health	14.583	28.473	.020	12.847	24.464	< 0.001
Impact on life	15.292	32.742	.032	19.458	32.600	< 0.001
Role limitations	8.250	38.649	.307	10.986	22.053	< 0.001
Physical limitation	8.333	28.448	.165	9.875	25.904	0.002
Social limitation	.083	20.581	.984	4.403	25.610	0.149
Personal relationships	-4.714	21.084	.418	8.872	24.369	0.029
Emotions	3.667	18.253	.335	11.653	20.147	< 0.001
Sleep/Energy	-1.458	14.623	.630	6.690	24.404	0.024
Incontinence	3.000	12.487	.251	3.014	14.733	0.087
Total KHQ	52.333	165.817	.136	84.111	136.806	< 0.001

In other subgroup analysis, the non-central obesity group showed no improvement in total IPSS, IPSS QoL, and total KHQ (Table 4). Among the KHQ domains, the non-central obesity group showed improvement only in general health (P = 0.020) and impact on life (P = 0.032). The central obesity group showed improvement in total IPSS, IPSS QoL, and total KHQ (Table 5). Among the KHQ domains, the central obesity group showed improvement in general health P < 0.001), impact on life (P < 0.001), role limitations (P < 0.001), physical limitation (P = 0.002), personal relationships (P = 0.029), emotions (P < 0.001), and sleep/energy (P = 0.024). The comparison of improvement of IPSS and KHQ revealed that central obesity group showed significant greater improvement in total IPSS, impact of life, and sleep/energy (Table 5).

**Table 5:** Comparison of the differences in IPSS and KHQ according to BMI and waist circumference

	**BMI < 23**	**BMI ≥ 23**	**P value**	**WC ≤ 90**	**WC > 90**	**P value**
IPSS questionnaire						
∆Total IPSS	3.37±6.17	6.00±7.66	0.041	2.87±7.18	4.30±6.38	0.028
∆IPSS QoL	0.96±1.52	1.00±1.22	0.830	0.79±1.79	1.02±1.34	0.231
KHQ questionnaire						
∆General health	10.71±20.26	14.00±26.71	0.286	14.58±28.47	12.84±24.46	0.321
∆Impact on life	17.76±31.12	20.06±37.19	0.052	15.26±32.53	19.42±32.46	0.028
∆Role limitations	10.61±27.10	9.48±27.05	0.564	8.30±38.54	11.06±22.11	0.356
∆Physical limitation	3.16±35.06	11.28±23.50	0.032	8.30±28.54	9.91±25.93	0.068
∆Social limitation	4.20±22.84	3.16±27.08	0.347	4.01±20.62	4.39±25.64	0.780
∆Personal relationships	5.71±27.96	5.39±22.24	0.561	5.09±22.05	4.71±28.32	0.814
∆Emotions	9.47±19.19	10.57±22.87	0.706	10.91±18.41	11.76±20.14	0.415
∆Sleep/Energy	3.98±21.63	7.11±25.48	0.031	3.21±14.61	6.68±24.23	0.035
∆Incontinence	19.69±24.66	18.35±24.96	0.381	18.43±24.99	19.72±24.64	0.451
∆Total KHQ	75.20±134.25	79.87±179.59	0.061	52.47±165.90	84.14±136.77	0.071

Adverse events were reported in 9 patients (6.0%) and included dizziness (3.3%), postural hypotension (1.3%), and gastric discomfort (1.3%).

## Discussion

When obese patients with BPH have LUTS, a larger prostate is presumed to be one of the causes. The prostate grows faster in obese than in non-obese patients [26-28].

Many clinical studies have focused on prostate size or PSA in obese patients, but few studies have focused on the treatment outcome, including QoL, in obese patients. Although several studies have used the IPSS-QoL index to present the treatment outcome in obese patients [14,29,30], the IPSS-QoL is not a disease-specific questionnaire. Rather, it represents the state of generalized HRQoL in men with LUTS.

The most prominent feature of our study was use of the KHQ questionnaire, a disease-specific HRQoL instrument for the evaluation of patients with LUTS, overactive bladder, and urinary incontinence [9,16-19]. The KHQ consists of nine domains. Seven of the domains include multiple questions including role limitations, physical limitations, social limitations, personal relationships, emotions, sleep/energy, and incontinence severity measures.

Our study revealed that comparison of improvement of IPSS QoL according to BMI and WC showed no significant difference. However, the comparison of improvement KHQ including impact on life, physical limitation, impact of life, and sleep/energy showed significant difference. Although improvement of total KHQ showed no significant difference, several domains of KHQ showed significant differences.

Although some controversies exist regarding the relationship between the severity of LUTS and obesity, obese patients usually have more severe LUTS than non-obese patients [29,30]. Possible reasons for this phenomenon are lower testosterone concentrations, lower serum globulin-binding protein levels, and greater prostate volumes in obese patients [31], as well as increased estrogen levels and increased free and total estradiol concentrations [32,33].

Our data demonstrate that the baseline total IPSS showed no significant difference between the obese and non-obese groups or central obesity and non-central obesity groups. This is mainly due to our lower IPSS inclusion criteria of more than 8 points total on the IPSS. We lowered the criteria for the IPSS because patients scoring higher on the IPSS could bias the results by showing more satisfaction with treatment.

Our data showed a more favorable treatment outcome in obese patients and those with central obesity. Although an improvement in the total IPSS was demonstrated in both obese and non-obese patients, the improvement in the total IPSS was only demonstrated in patients with central obesity. Improvement in total KHQ scores was demonstrated only in obese patients and patients with central obesity.

Greater estrogen levels in old age and obesity induce lower testosterone levels and may affect prostate cell growth [32]. Enlarged adipose tissue can secrete numerous hormones and proteins that influence fat metabolism [34,35], and leptin stimulates the cellular proliferation of BPH [36,37].

Besides an enlarged prostate, obese patients have increased sympathetic tone, which is suspected to be the main reason alpha blockers could have a greater impact in obese patients. Increased sympathetic tone can result in LUTS and subjective voiding complaints [19]. In particular, abdominal obesity increases the estrogen-to-androgen ratio and may increase sympathetic nervous system activity, both of which are known to influence the development of BPH and the severity of LUTS [18]. In our study, obese patients showed improvement in 5 domains of the KHQ, but central obesity patients showed improvement in 7 domains of the KHQ. This result implies that obesity, especially central obesity, which is regarded as visceral obesity, could impact treatment outcomes. The main reason for this result is that obesity, and especially visceral adiposity, is closely related to sympathetic overactivity [38,39]. The whole body norepinephrine spillover rate is known to be quantitatively linked with waist circumference [17,31], and sympathetic nerve firing rates measured by microneurography have been reported to be 55% higher in men with visceral adiposity than in men with only subcutaneous obesity.

Sympathetic activity is increased in obese men and may be related to BPH/LUTS by a common link between norepinephrine and alpha-1 adrenoceptors [38,39]. Although general improvement of LUTS and KHQ were observed in this study, the obesity and central obesity groups showed more improvement in the IPSS and KHQ.

This study has several limitations. First, the study design has a relatively short follow-up period. However, 8 weeks is sufficient to judge the clinical outcome of alpha blockers. Moreover, the KHQ is a relatively long questionnaire and takes a long time to perform, which reduces patient compliance. Although the acquisition of questionnaire was performed by one special interviewer, a lot of patients showed negative response to the KHQ in our study. The most common reason for this negative response is that it takes quite a long time. This is why we did not perform an additional KHQ at 4 weeks. Second, this study has a relatively small size. However, considering the increasing prevalence of concomitant medical diseases, the availability of eligible patients is limited. This was a pilot; a preliminary study, and subsequent prospective study is needed.

## Conclusions

Obese or central obesity patients with LUTS showed a better health-related quality of life than non-obese or non-centrally obese patients with LUTS after alpha-adrenergic medication. Therefore, alpha-adrenergic medication can be recommended to obese patients with LUTS preferentially for the improvement of quality of life.

## Competing interest

The authors declare that they have no competing interest.

## Authors’ contributions

JHK and YSS contributed with the conception and design of the study and drafted the manuscript, JHK, HC, SHY, SWD, WJY, BWY, JHY and MJK collected data, SSK performed the statistical analyses, and ESS, HJL, ISL, YSS have contributed on the critical revision of this manuscript. All authors read and approved the final manuscript.

## Pre-publication history

The pre-publication history for this paper can be accessed here:

http://www.biomedcentral.com/1471-2490/14/60/prepub
